# One step acid activation of bentonite derived adsorbent for the effective remediation of the new generation of industrial pesticides

**DOI:** 10.1038/s41598-020-76723-w

**Published:** 2020-11-19

**Authors:** Siti Fairos Ab Shattar, Nor Azazi Zakaria, Keng Yuen Foo

**Affiliations:** River Engineering and Urban Drainage Research Centre (REDAC), Engineering Campus, Universiti Sains Malaysia, Seri Ampangan, 14300 Nibong Tebal, Penang Malaysia

**Keywords:** Chemical engineering, Environmental chemistry, Green chemistry

## Abstract

Herein, the facile one step acid activation of bentonite derived functionalized adsorbent (AB) for the effective remediation of both ionic and non-ionic secondary pesticides, ametryn and metolachlor has been attempted. The surface characteristics of AB were examined by the nitrogen adsorption–desorption analysis, scanning electron microscopy (SEM), and Fourier Transforms Infrared (FTIR) Spectroscopy. The adsorptive behavior was evaluated with respect to the effect of contact time, initial concentrations and solution pH. The equilibrium data were fitted to the Langmuir, Freundlich and Temkin isotherm models, while the adsorption kinetic was analyzed using the pseudo-first order and pseudo-second order kinetic equations. Thermodynamic parameters including the standard enthalpy change (Δ*H*°), standard entropy change (Δ*S*°), and Gibbs free energy change (Δ*G*°) were established. Thermodynamic analysis illustrated that the adsorption process was feasible and exothermic in nature, while the characterization findings verified the alteration of FTIR bands, and a high specific surface area of 464.92 m^2^/g, with a series of pores distributed over the surface. Equilibrium data was best confronted to the pseudo-second order kinetic model, while the adsorptive removal of ametryn and metolachlor onto AB was satisfactory described by the Langmuir isotherm model, with the monolayer adsorption capacities for ametryn and metolachlor of 2.032 and 0.208 mmole/g respectively. The findings outlined the potential of the newly develop AB for the on-site treatment of pesticide polluted water.

## Introduction

Ametryn is an ionic pesticide of the *s*-triazine family, with a chemical structure of the aromatic hexamer ring. The specific action of ametryn on plants is the inhibition of photosynthesis process, particularly at the photosystem II (PSII), leading to the blockage of electron transport by displacing plastoquinone from the unique binding sites on the protein sub-unit, resulting in chlorosis (leaf yellowing) and tissue necrosis^1^. On the contrary, metolachlor is a non-ionic pesticide from the chloroacetanilide group that contain a chiral carbon atom, and usually exists as a pair of enantiomer. Metolachlor performs its action through the inhibition of cell growth to prevent the synthesis of long chain fatty acids (protein synthesis). Considerable studies have demonstrated that these pesticides could be up taken by the plant roots, and accumulate in the apical meristems to generate mutagenic and carcinogenic damages to the enzymatic systems and deoxyribonucleic acid (DNA). The biotransformation of these xenobiotics in plants could form the conjugates that are polymerized, to be incorporated into the plant structural components in such a way that these reactive intermediates, active oxygen species or the final metabolites would produce detrimental impacts, and secondly, they could be conjugated and stored in plants^2^. These xenobiotic metabolites could induce oxidative stress, a promoter of the cellular pathway associated with the onset, or progress of a variety of diseases: atherosclerosis, cancer, psoriasis, Alzheimer, hypertension and heart-liver failure^3,4^. During the reaction, the reactive species and these derivatives may attack lipids, proteins and nucleic acid molecules to cause metabolic alterations and cellular death. This has attested to a growing exploration for a new mitigative strategy for the effective control of these heavily polluted pesticides. Of major interest, adsorption process has been found to be superior to other techniques in terms of the low initial cost, high flexibility, ease of operation, and wider capability for the removal of a broad range of toxic water pollutants.

**Figure 1:**
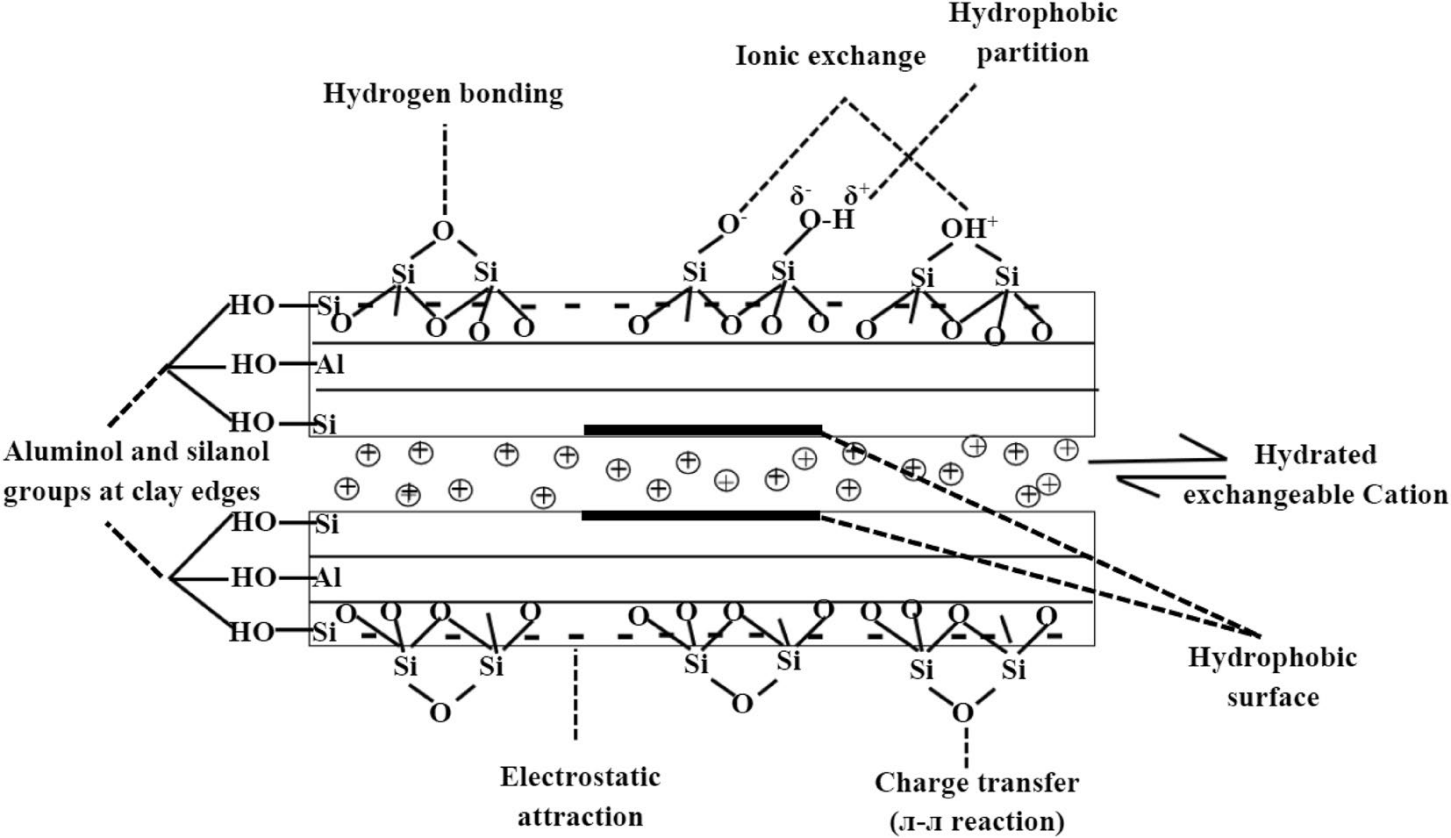
Probable interactive mechanism of ametryn and metolachlor onto AB.

Within this framework, bentonite, the dual-charge phyllosillicates particles originating from the alteration of glassy substances of igneous origin, with a high specific surface area of 20 to 130 m^2^/g, has found numerous applications in the ceramics, adhesives, catalysts, desiccants, cosmetics and pharmaceutical industries^5,6^. Its structure is formed by repetition units along the basal axis, composed of a central sheet of aluminum oxide (Al_2_O_3_) in octahedral geometry between two sheets of silicon oxide (SiO_2_) in tetrahedral geometry. The sheets are attracted to each another through the exchangeable cation balance (Na^+^, Ca^2+^, Mg^2+^, and K^+^) in their structure by electrostatic force^7^. The unique features of bentonite is the particle may develop a permanent and variable charge on the different surfaces of the same particle. The siloxane ditrigonal cavity of the phyllosilicate siloxane surfaces could form a localized permanent negative charge as a result of isomorphic substitutions in their internal crystalline structures. The magnitude of this permanent negative charge does not depend on the pH of a solution. In contrast, the edges of Fe/Al oxides have a surface reactive groups with amphoteric properties, resulting from the protonation and dissociation of Al–OH and Si–OH superficial functional groups, positively charged under acidic conditions or negatively charged under basic condition. The plausible mechanism/interaction for the adsorption of pesticide onto the clay is depicted in Fig. 1^8–10^. The wide versatility, naturally abundance, low cost, wide range of industrial uses, and their propensity to be chemically and physically modified, are the major driving force of the technological needs^11^. The wide scale implementations of bentonite however, is deteriorated by the presence of a variety of non-clay impurities that might influent negatively to shade its interesting key-properties intended for special purposes. In particular, bentonite purification via mechanical, thermal and chemical treatments often turn out to be necessary. Among these procedures, chemical modifications by acid or alkaline activation are usually expected to produce pronounce implications^12^.

Acid activation is a modification technique that aims to produce partial dissolution of the adsorbent to enhance the adsorptive properties, surface crystallinity, functionalities, specific surface area, and selectivity for different adsorbates. It is usually conducted for a total dissolution of undesired non-clay components and controlled attack, particularly for the octahedral layer to generate a more acidic Si-rich phase. Magnesium and to a lesser extend, aluminium are the most readily removed elements during acid modification. Similarly, bentonite activation is a complex phenomenon that involves the substitution of exchangeable cations, Al^3+^, Mg^2+^, and Fe^2+^ against protons from the octahedral sheet, resulting in the improvement of bleaching effectiveness, greater hydrophobicity and porosity structure, strongly protonated clay mineral surface, increase in specific surface area from the original 20–130 m^2^/g to higher than 200 m^2^/g, and generate higher affinity to the organic molecules such as ametryn and metolachlor^13^. In this sense, the present work was carried out to shed light on the feasibility of acid modified bentonite (AB) for the adsorptive treatment of ionic ametryn and non-ionic metolachlor, the second generation of industrial pesticide contaminants from the aqueous solution. Structural, functional and surface chemistry of the prepared adsorbent were evaluated. The effects of contact time, initial concentration, and solution pH on the adsorption performance were examined. Moreover, the adsorption isotherms, kinetics and thermodynamics analysis were elucidated.

## Materials and methods

### Modified bentonite

Bentonite, supplied by Sigma-Aldrich was applied as the raw precursor in this work. The acid modification was conducted by properly mixing bentonite with 8 M of nitric acid at 75 °C and 100 rpm. The modification process was terminated by the addition of a large amount of de-ionized water. The modified sample was rinsed and washed until the washing solution reached to the neutral pH. This newly prepared sample (AB) was dried at 60 °C for 24 h, and stored in an air-tight desiccator for further use.

### Adsorbate

Pure analytical-grade pesticides, ametryn and metolachlor were selected as the model pesticides in this study. The selected physico-chemical characteristics of ametryn and metolachlor are reported in Table 1. The standard stock solution was prepared by dissolving the parental compounds in a background solution of 0.01 M CaCl_2_ to keep the ionic strength unchanged, while the working solutions with the desired concentrations were prepared by a series of successive dilutions. The concentration range of 0.11–0.66 and 0.18–1.41 mmole/L, was selected in the adsorption test of ametryn and metolachlor, respectively, according to the solubility test.

**Table 1:** Physicochemical properties of ametryn and metolachlor.

Properties	Ametryn	Metolachlor
Scientific name	*N*-ethyl-*N*′-(1-methylethyl)-6-(methylthio)-1,3,5-triazine-2,4-diamine	*N*-(2-ethyl-6-methylphenyll-*N*-(2-methoxy-l-methylethyl) acetamide
Molecular formula	C_9_H_17_N_5_S	C_15_H_22_ClNO_2_
Molecular weight (g/mole)	227.3	283.3
Water solubility (mg/L)	200	488
*K*_oc_*	100–930	121–309
Vapour pressure (Pa)	3.7 × 10^–4^	4.2 × 10^–3^
Log *K*_ow_*	2.63	2.90
p*K*_a_*	4.1 (base)	Non-ionised

*K*_oc_* = The amount of chemical substance adsorbed onto adsorbent per amount of water.

*K*_ow_* = The ratio of the concentration of a chemical in *n*-octanol and water at equilibrium at a specified temperature.

p*K*_a_* = The negative base-10 logarithm of the acid dissociation constant (*K*_a_) of a solution.

### Batch equilibrium studies

The batch adsorption experiments were conducted in a series of 250 mL Erlenmeyer flasks containing 0.2 g of adsorbent and 200 mL of pesticide solutions. The flasks were capped and agitated in an isothermal water bath shaker at 120 rpm and 30 °C. The effect of solution pH on the adsorption process was examined by regulating the solution pH from 2 to 12 by the addition of 0.1 M of hydrochloric acid (HCl) or sodium hydroxide (NaOH) solution. The solution pH was measured using a pH meter (Accumet XL200, Fisher Scientific).

The sample was collected at prescribed time intervals, and the concentration of pesticide was determined using a UV–vis spectrophotometer (Shimadzu-1800, Japan), at the maximum wavelength of 224 and 194 nm, respectively for ametryn and metolachlor. All samples were filtered using a syringe filter (Whatman 0.45 µm) prior to analysis as to minimize the interference of clay fine particles with the analysis. In each experiment, the initial characteristics were determined to reduce the background interferences. Each adsorption experiments were replicated three times at least, and their arithmetical averages were applied as the result. The adsorptive uptake of ametryn and metolachlor at time, *t*, *q*_t_ (mmole/g) and equilibrium, *q*_e_ (mmole/g), was calculated by:

1 $$ q_{{\text{t}}}^{{}} = \frac{{(C_{{0}}^{{}} - C_{{\text{t}}}^{{}} )V}}{W} $$

2 $$ q_{{\text{e}}}^{{}} = \frac{{(C_{0}^{{}} - C_{{\text{e}}}^{{}} )V}}{W} $$

where *C*_0_, *C*_t_, and *C*_e_ (mmole/L) are the liquid-phase concentrations of ametryn or metolachlor at initial, time *t* (h) and equilibrium, respectively. *V* (L) is the volume of solution, and *W* (g) is the mass of adsorbent used.

### Adsorption isotherm

The dynamic separation of solute from the solution depends on the equilibrium separation between two phases. The equilibrium is established when the amount of adsorbed solute is equal to the amount being desorbed, and the quantity of solute uptake is a function of concentration of adsorbate at a constant temperature, and the resulting function could be expressed by the adsorption isotherms, an invaluable tool for the optimization of the adsorption pathways, reliable prediction of surface properties and adsorption parameters, viable design of the adsorption system and quantitative comparison of adsorbent behavior for different adsorption systems^14^. Based on the assumption of the monolayer adsorption, Langmuir isotherm^15^ is derived where there is no interaction between molecules adsorbed on adjacent site, and the adsorption occurs at specific uniform location on the adsorbent surface. In other words, the adsorption takes place at a fixed number of accessible homogeneous surface that are identical, with no transmigration of adsorbates in the plane of the neighbouring surface. This assumption however is not entirely true for all sorption cases since many sorption processes involve multilayer and heterogeneous surfaces. This led to the development of Freundlich isotherm model^16^ that is expressed as multilayer adsorption where interaction of adjacent molecules is possible on heterogeneous surface, with interactions between the adsorbed molecules and different adsorption energies and characteristics. Temkin isotherm model^17^, however takes into account of indirect sorbate/sorbate interactions on sorption surfaces. It assumes that as the surface is loaded up with adsorbate, and the heat of sorption of all the adsorbate molecules in the adsorbed layer would decrease linearly rather than logarithmically with the coverage due to sorbate/sorbate interaction. The nonlinear form of Langmuir, Freundlich and Temkin isotherm models are given by:

3 $$ q_{{\text{e}}}^{{}} = \frac{{Q_{0}^{{}} K_{{\text{L}}}^{{}} C_{{\text{e}}}^{{}} }}{{1 + K_{{\text{L}}}^{{}} C_{{\text{e}}}^{{}} }} $$

4 $$ q_{{\text{e}}}^{{}} = K_{{\text{F}}}^{{}} C_{{\text{e}}}^{{{1/}n}} $$

5 $$ q_{{\text{e}}}^{{}} = B\ln (AC_{{\text{e}}}^{{}} ) $$

where *Q*_0_ (mmole/g) and *K*_L_ (L/mole) are the Langmuir isotherm constants related to adsorption capacity and energy of adsorption, *K*_F_ (mmole/g) (L/mmole)^1/*n*^ and 1/*n* are the Freundlich isotherm constant, and a measure of adsorption intensity, and *B* = *RT*/*b*_T_, with *b*_T_ (J/mole), *R* (8.314 J/mole *K*), *T* (*K*) and *A* (L/mole) are the heat of sorption, universal gas constant, absolute temperature and equilibrium binding constants. To sufficiently apply the theoretical assumptions of these mathematical equations, several error deviation functions have been adopted for the prediction of the goodness of fit, derived as:

6 $$ R_{{}}^{2} = \frac{{(q_{{\text{e,meas}}}^{{}} - \overline{q}_{{\text{e,calc}}}^{{}} )_{{}}^{2} }}{{\sum {(q_{{\text{e,meas}}}^{{}} - \overline{q}_{{\text{e,calc}}}^{{}} )_{{}}^{2} + (q_{{\text{e,meas}}}^{{}} - q_{{\text{e,calc}}}^{{}} )_{{}}^{{2}} } }} $$

7 $$ RMSD = \frac{{\sqrt {\sum\nolimits_{i = 1}^{n} {(q_{{{\text{exp}}}}^{{}} - q_{{\text{p}}}^{{}} )_{{}}^{2} } } }}{n - 1} $$

where *R*^2^ is correlation coefficient, *RMSD* is root-mean-square deviation, *n* is the number of data points, *q*_e,meas_, *q*_e,calc,_ and $\overline{q}$_e,calc_ are the measured, calculated and average mean of adsorptive uptake (mmole/g), and *q*_exp_ (mmole/g) and *q*_p_ (mmole/g) are the experimental and predicted adsorption capacity, respectively.

### Adsorption kinetics and thermodynamics

Adsorption kinetic provides an insight into the controlling mechanism of the adsorption process, which in turn governs mass transfer and the residence time^18^. The nature of the surface interaction between the solute molecules and surface binding sites can be either physical (physisorption), chemical (chemisorption) or a combination of both, governed by the interactive forces involved. Several chemical reaction kinetic models have been reported to describe these interactions, and the most widely applied chemical reaction kinetic models are the pseudo-first order and pseudo-second order kinetic equations. Both pseudo-first order and pseudo-second order reaction models assume that the adsorption process is pseudo-chemical reaction. The pseudo-first order kinetic model^19^, popularly known as the Lagergren rate expression, is generally described by the equation:

8 $$ \ln \left(\frac{{q_{{\text{e}}}^{{}} }}{{q_{{\text{e}}}^{{}} - q_{{\text{t}}}^{{}} }}\right) = \frac{{k_{{1}}^{{}} }}{2.303}t $$

where *k*_1_ (1/h) is the pseudo-first order kinetic rate constant. The pseudo-first order reaction assumes the rate of occupation of binding sites is proportional to the number of unoccupied sites on the sorbent surfaces. Contrary to the pseudo-first order equation, pseudo-second order equation^20^ predicts the behavior over the whole range of adsorption, where the rate of occupation of binding sites is proportional to the square of the number of unoccupied sites on the sorbent surfaces. For the boundary conditions of *q* = 0 at *t* = 0 and *q* = *q*_t_ at *t* = *t*, the Ho’s kinetic model is derived as:

9 $$ \frac{1}{{(q_{{\text{e}}}^{{}} - q_{{\text{t}}}^{{}} )}} = \frac{1}{{q_{{\text{e}}}^{{}} }} + k_{2}^{{}} t $$

where *k*_2_ (g/mmole h) is the pseudo-second order kinetic rate constant. The suitability of the kinetic model to describe the adsorption process was ascertained by the value of correlation coefficient, *R*^2^ and the normalized standard deviation, Δ*q* (%) derived as:

10 $$ \Delta q(\% ) = 100\sqrt {\frac{{\sum {\left[ {(q_{{{\text{exp}}}}^{{}} - q_{{{\text{cal}}}}^{{}} )/q_{{{\text{exp}}}}^{{}} } \right]^{2} } }}{n - 1}} $$

where *q*_exp_ (mmole/g) and *q*_cal_ (mmole/g) are the experimental and calculated adsorption capacities, respectively.

Thermodynamic is a critical aspect predicting the stability of the solid–liquid phase equilibrium, and is a basic requirement for the characterization of an adsorption system. The thermodynamic characteristics describe the surface changing process from the initial state to the final equilibrium and second, the alterating surface binding forces and energy under equilibrium conditions. The thermodynamic constants, specifically standard Gibbs free energy change (Δ*G*°), standard enthalpy change (Δ*H*°), and standard entropy change (Δ*S*°) were adopted to evaluate the possible phenomena taking place with the adsorption process, the isosteric heat of adsorption and solute–surface interactions. In this work, the values of enthalpy change (Δ*H*°), Gibbs free energy change (Δ*G*°), and entropy change (Δ*S*°) at the adsorption temperatures of 30, 40 and 50 °C were computed by:

11 $$ K_{{\text{d}}}^{{}} = \frac{{C_{{{\text{Ae}}}}^{{}} }}{{C_{{\text{e}}}^{{}} }} $$

12 $$ \ln K_{{\text{d}}}^{{}} = \frac{\Delta S}{R} - \frac{\Delta H}{{RT}} $$

13 $$ \Delta G = - RT\ln K_{{\text{d}}}^{{}} $$

where *K*_d_ is the distribution coefficient for the adsorption, *C*_Ae_ is the amount adsorbed on solid at equilibrium, *T* (*K*) is the absolute temperature, and *R* (8.314 J/mole *K*) is the universal gas constant.

### Adsorption mechanism

To verify the mechanism of the adsorption process, the intraparticle diffusion model, an empirically found functional relationship, proposed by Weber and Morris^21^ was applied to elucidate the governing steps of the adsorption process. According to the model, the adsorptive uptake of ametryn and metolachlor would proportionally to *t*^1/2^ rather than with the contact time *t*, defined as:

14 $$ q_{{\text{t}}}^{{}} = k_{{{\text{pi}}}}^{{}} t_{{}}^{{1/2}} + C_{{\text{i}}}^{{}} $$

where *k*_pi_ (mg/g h^1/2^) is the intraparticle diffusion rate constant, and *C*_i_ gives an idea about the thickness of the boundary layer. According to the model, if the intraparticle diffusion occurs, the *q*_t_ versus *t*^1/2^ will be linear, and intraparticle diffusion is the sole rate limiting step, if the plot passes through the origin. Otherwise, some other mechanism along with intraparticle diffusion may be involved.

To determine the actual rate-controlling step of the adsorption process, the sorption data were further analyzed using the theoretical equation given by Boyd et al.^22^, express by:

15 $$ B_{{\text{t}}}^{{}} = - 0.4977 - \ln (1 - F) $$

which *B*_t_ is the mathematical function of *F*, and *F* represents the fraction of solute adsorbed at time, *t* (h), given by:

16 $$ F = \frac{{q_{{\text{t}}}^{{}} }}{{q_{{\text{e}}}^{{}} }} $$

### Physical and chemical characterizations

Scanning electron microscopy, SEM (Zeiss Supra 35VP) analysis was carried out to evaluate the textural morphology of the raw bentonite and AB. The surface physical properties were characterized by Micromeritics ASAP 2020, using nitrogen (N_2_) as the adsorbate at 77 K, while the surface functional groups were detected by Fourier Transform Infrared (FTIR) Spectroscopy (Perkin Elmer-1600) from the scanning range of 4000–400 cm^−1^. The zero point of charge (*pH*_pzc_) was conducted in a set of Erlenmeyer flasks containing 50 cm^3^ of 0.01 M of sodium chloride (NaCl) solution, and the pH of the solutions was adjusted to a value from 2 to 12. 0.15 g of AB was added, and the solution pH was measured after 48 h under agitation. The *pH*_pzc_ is the point where the curve *pH*_final_-*pH*_initial_ = 0.

## Results and discussion

### Batch adsorption studies

#### Effect of contact time and initial concentration

**Figure 2:**
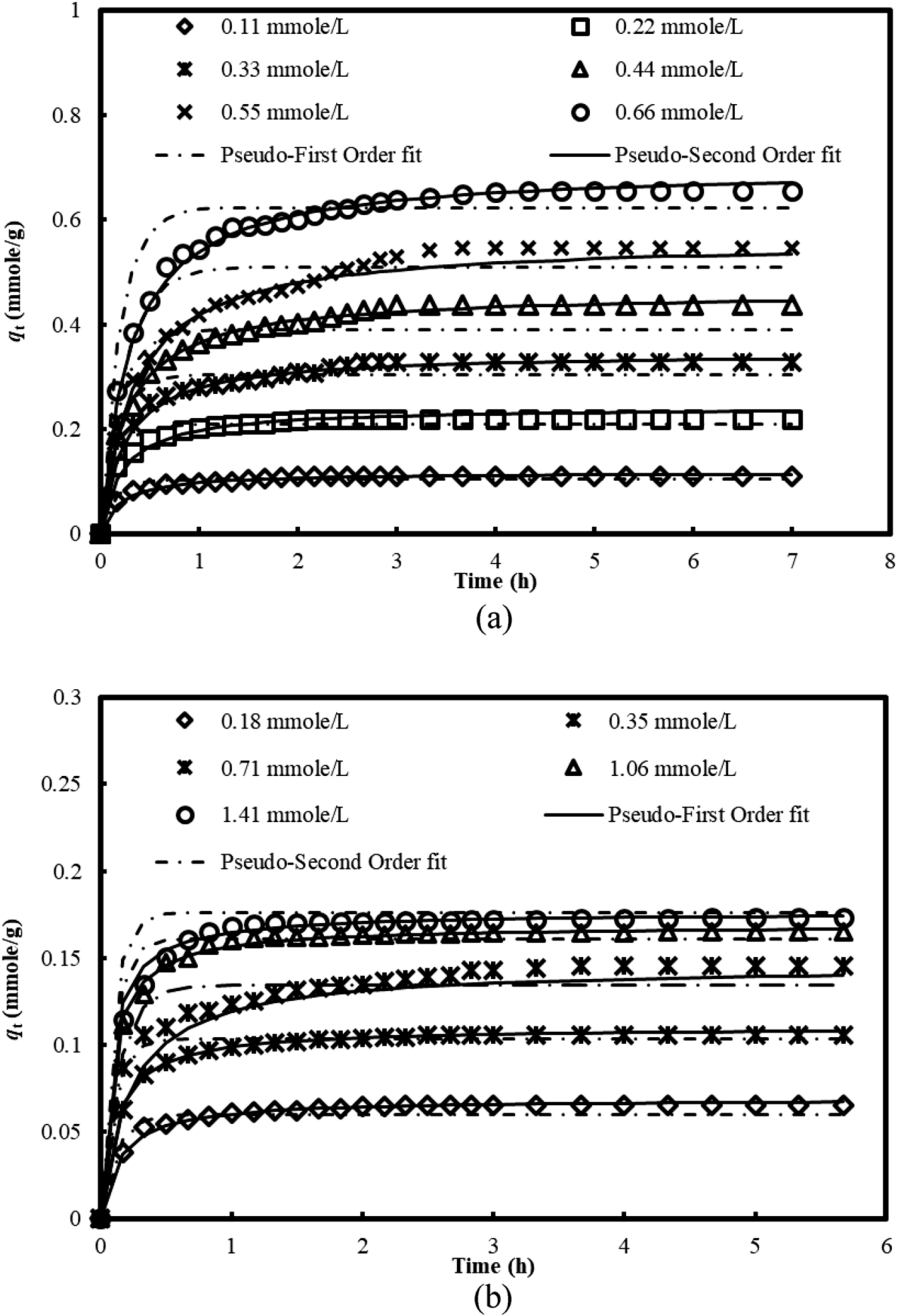
Effect of initial concentrations and contact time on the adsorptive uptakes of (**a**) ametryn and (**b**) metolachlor onto AB at 30 °C.

The effect of initial concentrations remains a crucial parameter for the quantification of concentration dependence on the rate of the adsorption process. Figure 2 presents the plots of pesticides uptakes, *q*_t_ (mmole/g) with respect to time, *t* at the initial concentrations of 0.11–0.66 mmole/L and 0.18–1.41 mmole/L, adsorbent dosage and operating temperature of 0.2 g/200 mL and 30 °C, for ametryn and metolachlor, respectively. Generally, the pesticide uptakes increased rapidly within the first 30 min, and thereafter the adsorption rate decreased gradually, and reached to plateau, to turn into the equilibrium stage. Near to the equilibrium state, only a slight increase of the adsorptive uptakes was observed, as the available binding sites for the pesticide’s entrapment were limited and, during the equilibrium stage, prolonging the contact time did not show any obvious changes of the adsorptive uptakes. This observation was mainly due to the great availability of surface binding sites to be assessed at the early stage. However, as the equilibrium approached, these readily vacant surface sites were gradually reduced, and the adsorption process turned lower, driven by the repulsive forces between the adsorbate molecules on the bulk and solid surfaces. At this stage, a dynamic equilibrium between the adsorbate molecules being adsorbed and desorbing from the adsorbent has been established. The amount of adsorbate being adsorbed onto the adsorbent at the equilibrium state is termed as equilibrium uptake, and the time required to achieve the equilibrium stage, is designated as equilibrium time^23^.

From Fig. 2, by increasing the initial concentrations of ametryn from 0.11 to 0.66 mmole/L and metolachlor from 0.18 to 1.41 mmole/L, the adsorption equilibrium (*q*_e_) increased from 0.11 to 0.66 mmole/g, and from 0.07 to 0.17 mmole/g, respectively. It was evident that increasing initial concentration showed a greater driving force, and mass transfer governing the adsorption process, resulting in the higher adsorptive uptakes of ametryn and metolachlor. It is clear from Fig. 2 that the adsorption equilibrium of ametryn and metolachlor onto AB has been completed within 4 and 2 h, respectively. The contact time required for the adsorption process was relatively short, indicating the real practicability for the on-site applications. The time profile of ametryn and metolachlor uptake is a single, smooth and continuous curve leading to saturation, suggesting possible monolayer coverage of ametryn and metolachlor onto the surface of AB. Comparison of the adsorptive findings illustrated that the adsorptive interaction between ametryn with AB was greater than metolachlor, mainly due to the non-ionic behavior of metolachlor, which is driven by the physical bonding. Conversely, the interaction, adsorptive removal of ametryn could be related to both ionic and non-ionic characteristics that implies both physical and electrostatic interaction.

### Effect of solution pH

**Figure 3:**
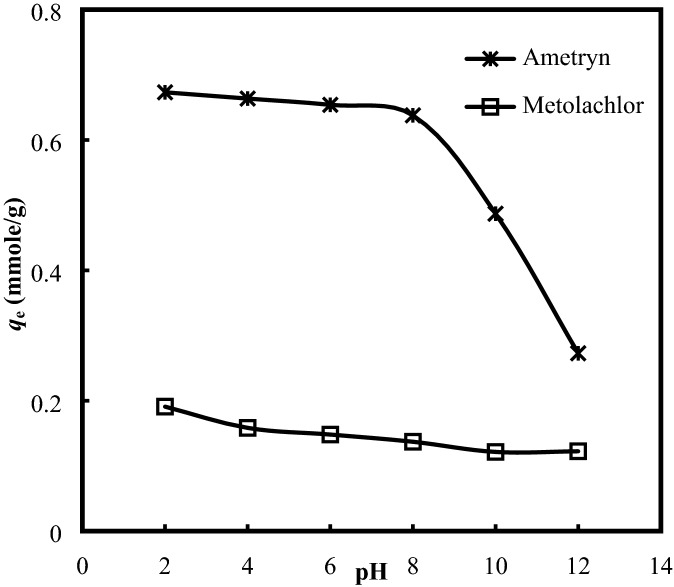
Effect of pH on the adsorptive uptakes of (**a**) ametryn and (**b**) metolachlor.

In general, ametryn can be adsorbed onto the clay minerals as both protonated and neutral species. The neutral form is adsorbed by relatively weak physical forces (hydrophobic partitioning, van der Waals forces and H-bonds), whereas the positively charged molecule is mostly adsorbed by cation exchanged, while the non-ionic metolachlor could be adsorbed to the clay surfaces through the formation of complex, an exchangeable cation and the clay surface, lead to the immobilization and activation. Figure 3 shows the effect of solution pH on the adsorptive uptakes of ametryn and metolachlor onto AB. The adsorptive removal of ametryn was highly dependent on the solution pH, as it would affect the surface charge and the ionization of the adsorbent surface and adsorbate molecules. It was found that decreasing the solution pH from 12 to 2 led to an increase in the adsorptive uptake of ametryn from 0.27 to 0.67 mmole/g (Fig. 3). As the solution pH decreased, the protonation of the ametryn took place, and the resulting cations were more easily to be adsorbed onto the negatively charged AB. This cationic or protonated form of ametryn is much stronger to be retained than the dissociated form of ametryn, that would hold tightly on the negatively charged clay minerals to resist the desorption process. Moreover, AB surface has a *pH*_pzc_ value of 2.37. At the pH above the zero point of charge (*PZC*), the surface of AB has a net negative charge, and the adsorption of anion is restricted by the electrostatic repulsive force. At the pH value lower than the PZC, the adsorption of anionic ametryn could be promoted by the electrostatic attraction to the positively surface charge of AB, via the anion exchange, cation bridging and ligand exchange mechanism. The preferential adsorption of ametryn at the acidic condition has been well documented by Ahmad et al.^24^, Yamane and Green^25^, and Yang et al.^26^.

On the contrary, the adsorptive uptake of metolachlor was almost unaltered by the variation of solution pH from 4 to 12 (Fig. 3). Generally, metolachlor possess weakly polar functional group, and the adsorptive characteristic is neither governed strictly by the non-polar nor polar functionalities, but relies on the ability for the displacement of water molecules on its’ surface to gain a higher entropy energy, that would favour the adsorption process. However, the slightly decrease of the adsorptive uptake of metolachlor from 0.19 to 0.16 mmole/g as the initial pH increased from 2 to 4 may be due to the presence of excess H^+^ ions, which have accelerated the removal of metolachlor in the aqueous solution^27^. At the very low solution pH values, the surface of adsorbent would be surrounded by the hydronium ions, to enhance the interaction with the surface binding sites by greater attractive forces to improve the uptake of the polar molecules. At the highly acidic pH, the surface of AB was positively charged, and this suggested that the main interactions between the adsorbent and metolachlor at pH < 3 is governed by the electrostatic force between the positively charged AB and the electron rich regions in the adsorbed molecules. At pH equal to *pH*_pzc_, the surface of AB was essentially neutral, weakening the interaction with metolachlor, to decrease the degree of adsorption. Similar trend was observed in the adsorption of metolachlor onto organohydrotalcites^28^ and lindane onto the Rhizopus oryzae derived biosorbent^29^.

### Adsorption isotherm

Adsorption isotherm is essential for the unique design and upscalling of the adsorption system^30^. The adsorption isotherm parameters obtained at different operating temperatures, and the corresponding *R*^2^ and *RMSD* are presented in Table 2. Comparison of the values of *R*^2^ and *RMSD* summarized in Table 2 ascertained that the Langmuir isotherm model showed a better suitability to describe the adsorption of ametryn and metolachlor onto AB, with the monolayer adsorption capacity for ametryn and metolachlor of 2.032 mmole/g and 0.208 mmole/g respectively. The applicability of the Langmuir isotherm model suggested the monolayer coverage of ametryn and metolachlor molecules on the outer surface of AB, with no energy or heat transfer within the adsorbing surface. A comparative evaluation of the monolayer adsorption capacities of ametryn and metolachlor onto different functionalized adsorbents are listed in Table 3. The adsorbent prepared in this work showed comparable performance as compared with previous researches as reported in the literature.

**Table 2:** Adsorption isotherm parameters for the adsorption of ametryn and metolachlor onto AB at 30 °C.

Adsorbate	Langmuir isotherm model			*RMSD*	Freundlich isotherm model			*RMSD*	Temkin isotherm model			*RMSD*
	*Q*_0_ (mmole/g)	*K*_L_ (L/mmole)	*R*^2^		*K*_F_ (mmole/g).(L/mmole)^1/*n*^	*n*	*R*^2^		*A* (L/mole)	*B*	*R*^2^	
Ametryn	2.032	101.92	0.998	0.0049	38.23	1.319	0.983	0.0071	2471.83	0.246	0.948	0.0164
Metolachlor	0.208	4.18	0.999	0.0003	0.618	2.769	0.949	0.0035	39.76	0.046	0.981	0.0015

**Table 3:** Comparative evaluation of monolayer adsorption capacities for ametryn and metolachlor onto different functionalized adsorbents.

Herbicide	Adsorbent	Activating agent	Monolayer adsorption capacity (mmole/g)	References
Ametryn	Bentonite	HNO_3_	2.03	This study
	Commercial AC		1.01	Yang et al.^26^
	Soil	Organic matter	(2.20–5.73) × 10^–4^	Sluszny et al.^31^
	Chalk	–	6.82 × 10^–3^	Wefer-Roehl et al.^32^
	AC-cloth (spectracarb 2225)	–	1.56	Ayranci and Hoda^33^
Metolachlor	Bentonite	HNO_3_	0.21	This study
	Activated charcoals	–	(4.94–5.65) × 10^–4^	Bosetto et al.^34^
	Wyoming bentonite	–	1.76 × 10^–4^	Nennemann et al.^35^
	Fly ash	–	3.53 × 10^–3^	Singh^36^
	Clay loam soil	–	3.53 × 10^–5^	Si et al.^37^
	Soil	Fly ash	0.06–0.10	Ghosh and Singh^38^
	Granular carbon	–	0.01	Kumar et al.^39^
	Periodic mesophorous organosilica	Benzene	7.41 × 10^–4^	Otero et al.^40^

The degree of suitability and fundamental practicability of the Langmuir isotherm model was further justified using a dimensionless constant^41^, separation factor (*R*_L_) expressed by:

17 $$ R_{{\text{L}}}^{{}} = \frac{1}{{1 + K_{{\text{L}}}^{{}} C_{0}^{{}} }} $$

which the value of *R*_L_ could indicate the sorption either to be unfavourable (*R*_L_ > 1), linear (*R*_L_ = 1), favourable (0 < *R*_L_ < 1), or irreversible (*R*_L_ = 0). The degree of the favourability is related to the irreversibility of the adsorption system, to provide a qualitative assessment of the adsorbent-adsorbate interactions. From Supplemental Figure 1, the *R*_L_ values for the adsorption of ametryn and metolachlor onto AB fall between 0 and 1, which implied that the adsorption of ametryn and metolachlor onto AB from the aqueous solutions was favourable under the conditions applied. The *R*_L_ values decreased as the initial concentration increased from 0.11 to 0.66 mmole/L, and from 0.18 to 1.41 mmole/L for ametryn and metolachlor, respectively, indicating the adsorption was favourable at the higher initial concentration range.

### Adsorption kinetics

Adsorption kinetic study is important for the prediction of the optimum conditions for the real practical applications^42^. The analysis expresses the solute uptake rate, which in turn govern residence time of the adsorption reaction. The pseudo-first order and pseudo-second order kinetic equations are the most well-liked models for the quantitative evaluation of the adsorption process. The pseudo-first order kinetic model depends mainly on the adsorbate concentration, and provides a good description of the adsorption of contaminants at very low concentrations, while the pseudo-second order kinetic model was derived from the adsorption process, where the rate-controlling step is driven by the ion exchange interaction.

To verify the suitability of the fitting model, the kinetic data was evaluated by the determination of correlation coefficient (*R*^2^) and the normalized standard deviation, Δ*q* (%) as presented in Supplemental Table 1. Result (Fig. 2) showed that the pseudo-first order kinetic model fitted weakly to the experimental data, with the low *R*^2^ of 0.913 to 0.996 and high Δ*q* values of 3.43% to 16.71%. On the contrary, the pseudo-second order model could adequately explain the experimental data, with great agreement between the experimental and theoretical *q*_e_ values, verified by the highest *R*^2^ of greater than 0.99, and the lowest Δ*q* (%) values range of 1.37% to 10.05%. Similar observations have been deduced for the adsorption of 2,4-dichlorophenoxyacetic acid (2,4-D) onto oil palm frond derived activated carbon^43^ and calcined Zn-Al-Zr layered double hydroxide^44^. The findings were in good agreement with the adsorption of metalaxyl and tricyclazole onto the natural clays derived adsorbent^45^ and fenarimol onto the Fe_2_O_3_-palygorskite nanoparticles^46^. This result ascertained the suitability of pseudo-second order kinetic model to describe adsorption process, based on the assumption that chemisorption is the rate limiting step, that involved valency forces through electron sharing or exchange between the adsorbate molecules and the clay based adsorbent.

### Adsorption mechanism

Adsorption mechanism can generally be described by four consecutive rate controlling steps, namely external mass transfer (transport from the bulk solution to the adsorbent surface), film diffusion (diffusion across the liquid film from the adsorbent surface), intraparticle diffusion (pore diffusion, surface diffusion or a combination of both), and surface interaction on the adsorbent active sites. It is commonly known that the adsorption process is mainly governed by the intraparticle diffusion mechanism. This rate limiting step can be qualitatively determined by analyzing the kinetic data using the Weber-Morris model. In brief, the plot of *q*_t_ versus *t*^0.5^ could be multi-linear, that indicates two or more steps may involve during the adsorption process. If the intra-particle diffusion is the only rate-controlling step, the plot would pass through the origin. Otherwise, more than one steps could have affected the adsorption process. From Supplemental Figure 2, the first sharper region was the external diffusion step that has been completed within 1 h. The slow gradually adsorption process observed in the second region was ascribed to the diffusion of adsorbate molecules into the interior surface of the solid adsorbent. The third region that showed a plateau was the equilibrium stage, with intra-particle diffusion as the main controlling mechanism. The dissimilarity of the mass transfer in the initial and final stages of the adsorption process implied the presence of multi-linearity, with intra-particle diffusion was not the only rate-limiting mechanism. Similar findings have been reported for the adsorption of atrazine and metolachlor onto the natural soil^47,48^.

The values of *K*_pi_, *C*_i_, and *R*^2^ determined from the three regions are listed in Supplemental Table 2. A three-stage multi-linear sorption process represents: (a) 1st stage (*k*_p1_) was driven by the boundary layer diffusion of the sorbate molecules; (b) 2nd stage (*k*_p2_) was the gradual intraparticle diffusion and (c) 3rd stage (*k*_p3_) was attributed to the final equilibrium of the sorption process. Increasing the bulk pesticides concentration showed an enhancement of the pore diffusion rate, *k*_pi_, indicative of greater driving force for the adsorption process. By interpreting the values of intercept *C*_i_, the information about the thickness of the boundary layer and/or the resistance to the external mass transfer can be deduced. Larger the intercept, greater is the contribution of the surface sorption in the rate-limiting step. The values of *C*_i_ were found to increase gradually from 0.083 to 0.643, and from 0.050 to 0.170 by increasing the initial concentrations from 0.11 to 0.66 mmole/L and from 0.18 to 1.41 mmole/L for ametryn and metolachlor, respectively, illustrating increase of thickness of the external boundary layer, and the external mass transfer resistance. The Weber-Morris model is a viable tool to elucidate an initial understanding of the diffusion step of an adsorption process. Therefore, the equilibrium data has been further fitted to the Boyd equation, for the verification of pore and film diffusion mechanism, and identification of the slowest steps throughout the adsorption process. Accordingly, the slowest or rate limiting step could be rated as pore diffusion if the plot *B*_t_ versus *t* is linear, and pass through the origin. Conversely, the adsorption process would be mainly governed by film diffusion mechanism. From Supplemental Figure 3, the acquired curves did not pass through the origin, with the data scattering surrounding the curves. The presented results ascertained the adsorption system of this work could be affected primarily by the film diffusion controlling step.

### Adsorption thermodynamics

The three major thermodynamic parameters (Supplemental Table 3), standard enthalpy change (Δ*H*°), standard entropy change (Δ*S*°) and Gibbs free energy change (Δ*G*°) were determined from the slope and intercept of the van’t Hoff plot of ln *K*_d_ versus 1/*T*. Negative value of Δ*G*° indicated high feasibility of the treatment process, and spontaneous nature of the adsorption interaction, with a high preference of ametryn uptake onto the prepared AB. Increasing the operating temperature from 30 to 50 °C showed a gradually increase of Δ*G*° values from − 12.74 to − 10.04, and − 0.09 to 3.69 kJ/mole for the adsorption of ametryn and metolachlor, respectively, indicating exothermic nature of the adsorptive interaction. The greater the Δ*G*° value at higher operating temperatures is related to the surface tension of the prepared adsorbent minimizing their respective surface area. As a result, an extra surface free energy is required to extent the surface area by stretching or distorting their respective surface. The result could be attributed to the enhancement of the solubility and dissipation rate of the adsorbate molecules, and desorption of the adsorbate molecules from the solid adsorbent, due to the dislodgement of the adsorptive forces.

Increasing the operating temperatures would also induce the weakening of the adsorptive forces between the surface-active sites with the pesticide molecules, and between the adjacent pesticide molecules on the adsorbed phase. The findings were in according to the negative Δ*H*° of − 42.08 kJ/mole and − 57.18 kJ/mole for ametryn and metolachlor, respectively. The positive value of Δ*S*°, 196.14 J/mole *K* showed the high affinity of AB for ametryn, and the increasing randomness at the solid-solution interface during the adsorption process. Contradict to ametryn, metolachlor adsorption onto AB showed the negative value Δ*S*° of − 187.94 J/mole *K*, illustrated the attachment process was enthalpy driven, associated with mild dehydration of the surface structural changes of the solid adsorbent. The process was rather an addition reaction than a replacement reaction. Similar observation has been drawn by Gładysz-Płaska et al.^49^ and Sathishkumar et al.^50^ for the adsorption of chromium (VI) and phenol onto the HDTMA modified natural red clay, and adsorption of 2,4-dichlorophenol onto the palm pith derived activated carbon.

### Textural and surface characterizations

**Figure 4:**
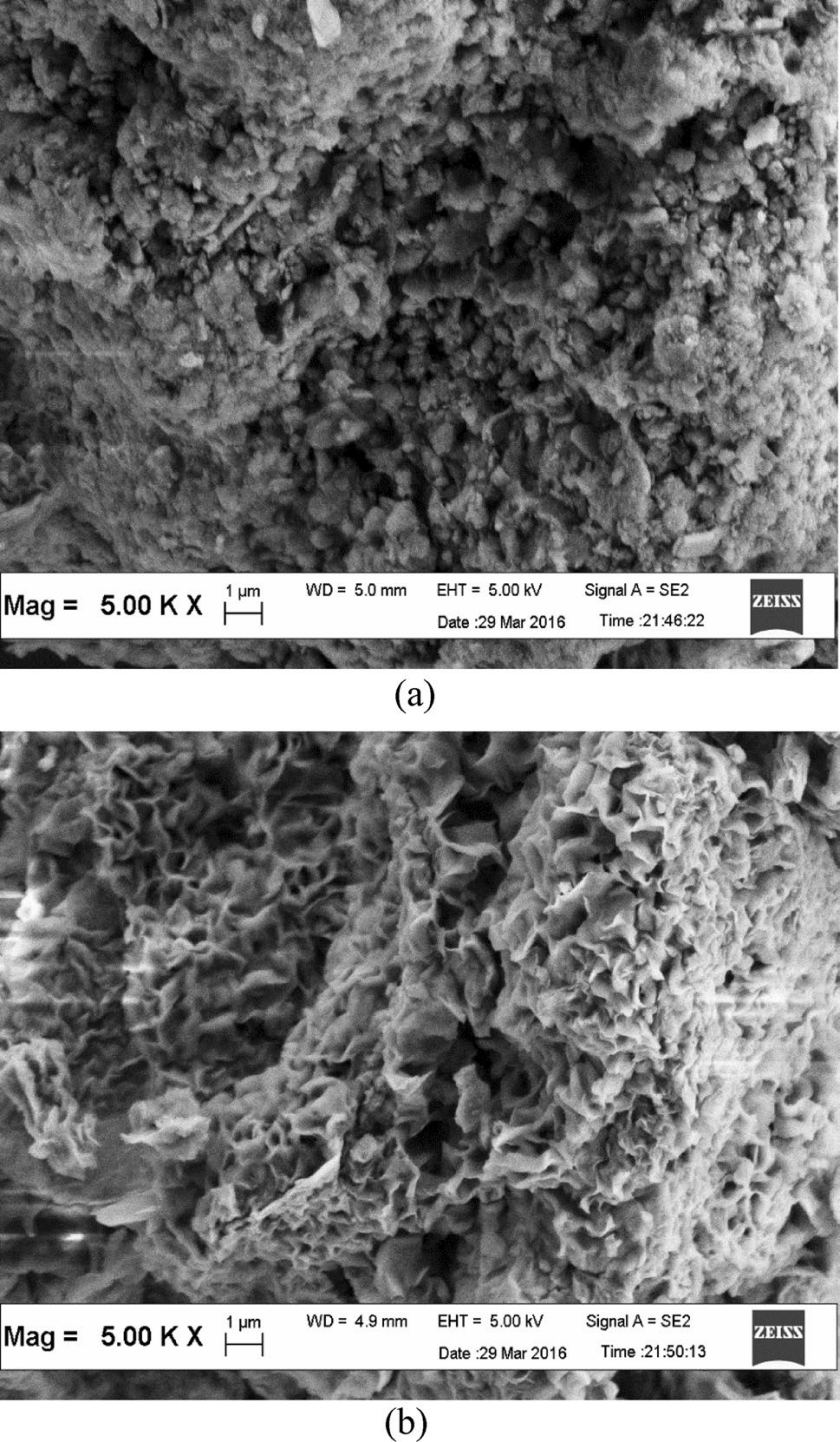
Scanning electron micrographs of (**a**) bentonite and (**b**) AB.

**Figure 5:**
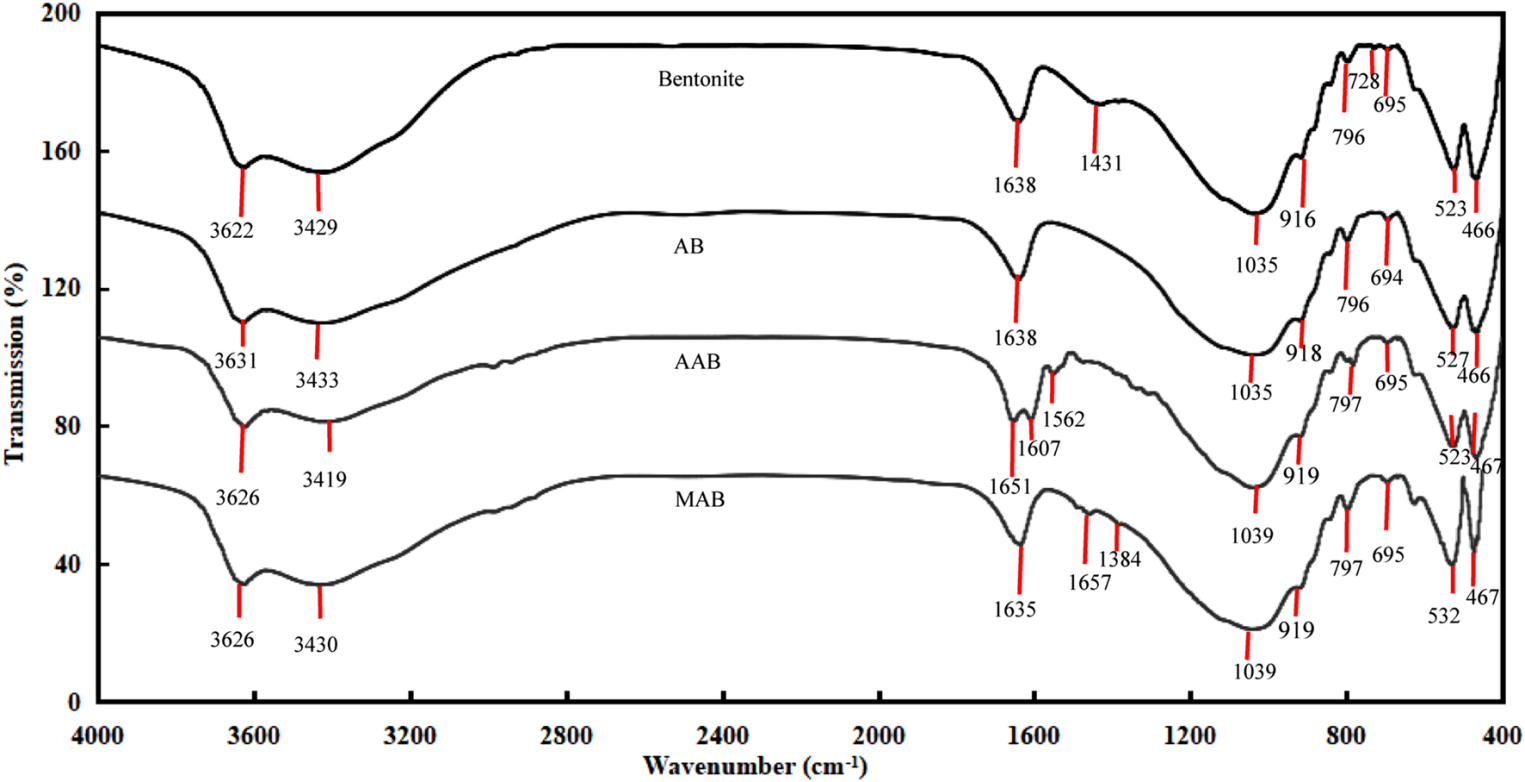
Fourier Transform Infrared Spectra of bentonite, AB, ametryn loaded AB and metolachlor loaded AB.

The morphological features of the natural bentonite and AB are displayed in Fig. 4. The surface morphology of the raw bentonite demonstrated a dense and compact structure, dispread by the deposited tarry substances. However, the newly prepared AB showed a well-organized and uniform porosity, with a series of micro-passage distributed around the surface. The pores developed over the surface indicated good possibility for the pesticides to be adsorbed. Similar observation was reported by Majid and Azimi^51^ and Toor et al.^52^ on the hydrochloric acid treated bentonite. The Fourier Transform Infrared Spectra of the raw bentonite, AB, ametryn loaded AB (AAB) and metolachlor loaded AB (MAB) is presented in Fig. 5. The signals at 3631–3622, 3433–3419 and 1651–1635 cm^−1^ are ascribed to the stretching and vibrations hydration of hydroxyl molecules, while the intensive peaks at 1039–1035, 919–916, 796–797, 728, 695–694, 532–523 and 467–466 cm^−1^ are assigned to the in plane Si–O, Al–OH–Al vibrations, Al–Mg–OH bending, quartz or/and amorphous silica admixture, OH bending, Si–O–Al deformation and Si–O–Si bending present in the bentonite derived adsorbent. During the modification process, the protons would penetrate into the bentonite layers to attack the OH groups, leading to the alteration of OH vibration and octahedral cations. The resulting dehydroxylation is connected to the successive release of the central atoms from the octahedral surface, and the removal of Al from the tetrahedral sheets. Meanwhile, a gradual transformation of the layered tetrahedral sheet to a three-dimensional framework proceeds, where changes in the characteristics of hydroxyl groups and the silicate anions could be observed, detected by the alteration of FTIR bands. These transformations have assisted the formation of amorphous silica to provide a number of surface binding site for the adsorption process. In contrast, the intensities detected at 1657, 1607, 1562 and 1384 cm^−1^ at the FTIR spectrum for ametryn and metolachlor loaded AB were resulted from the pesticide vibrations of the ametryn or metolachlor adsorption onto AB. Similar phenomenon was reported by Davies and Jabeen^53^, Giroto et al.^54^ and Wei et al.^55^.

The nitrogen adsorption–desorption curves for the raw bentonite and AB are displayed at Supplemental Figure 4. The hysteresis loop featured a hybrid type I–II classification, associated with the capillary condensation in mesopores. The rising parts of the nitrogen isotherm at high relative pressure demonstrated the presence of microporous structure of the tested adsorbents. The surface physical parameters of the clay-based adsorbents are listed in Supplemental Table 4. The BET surface area, Langmuir surface area and total pore volume for the raw bentonite and AB were identified to be 120.35 m^2^/g, 151.75 m^2^/g, and 0.155 cm^3^/g, and 464.92 m^2^/g, 558.48 m^2^/g, and 0.239 cm^3^/g, respectively, resulted from the dissolution of impurities, and substitution of exchangeable cations with hydrogen ions to enhance the specific surface area. Similar phenomenon has been reported by Arfaoui et al.^56^ and Srasra et al.^57^ on the hydrochloric acid, nitric acid and sulfuric acid treated bentonite. The representative pore size distribution of the raw bentonite and AB is depicted in Supplemental Figure 5. According to the classification of International Union of Pure and Applied Chemistry (IUPAC), the pores diameter could be classified into three major categories: macropores (d > 50 nm), mesopores (2 < d < 50 nm) and micropore (d < 2 nm). The graph detected the sharpest peak at the pore diameter between 20 to 100 Å, with an average pore size of 46.67 Å and 63.48 Å, respectively for the raw bentonite and AB, consequent of the disintegration of the crystal structure and intraparticle space collapse to transform part of the micropores into the mesoporous structure. The results were in according with those reported on the smectite modification process^58^.

## Conclusion

The present investigation demonstrated the promising role of AB for the successive treatment of both ionic and non-ionic secondary pesticides, ametryn and metolachlor. The time profile for the adsorption of ametryn and metolachlor onto AB was relatively short, indicating the high economic feasibility for the real practical application. Equilibrium data were satisfactory fitted to the Langmuir isotherm model, with the maximum monolayer adsorption capacities for ametryn and metolachlor of 2.032 mmole/g and 0.208 mmole/g, respectively. The adsorption kinetic was best described by the pseudo-second order kinetic equation, which suggested that the adsorption rate was highly dependent on the availability of the adsorption sites rather than by the changing concentrations. Thermodynamic study ascertained the spontaneous nature of the treatment process. The research findings verified the potential of AB as the next-generation of functionalized adsorbent for the preventive treatment of the multi-pronged pesticides contaminated wastewater.

## Supplementary information

Supplementary Information.
