# Supplementary material for: One step acid activation of bentonite derived adsorbent for the effective remediation of the new generation of industrial pesticides
Source: Sci Rep. 2020 Nov 19;10:20151. doi: 10.1038/s41598-020-76723-w (PMC7677388; doi:10.1038/s41598-020-76723-w)
Supplement: Supplementary file 1 — Supplementary Information. [file 41598_2020_76723_MOESM1_ESM.doc]

**One step activation of bentonite derived adsorbent for the effective remediation of the new generation of industrial pesticides**

**Authors’ name and affiliations:**

Siti Fairos Ab Shattar (S.F.A. Shattar)

River Engineering and Urban Drainage Research Centre (REDAC)

Higher Institution Centre of Excellence (HiCoE)

Engineering Campus, Universiti Sains Malaysia, Seri Ampangan

14300 Nibong Tebal, Penang, Malaysia

Email: [ctfairosz89@yahoo.com.my](mailto:ctfairosz89@yahoo.com.my)

Nor Azazi Zakaria (N.A. Zakaria)

River Engineering and Urban Drainage Research Centre (REDAC)

Higher Institution Centre of Excellence (HiCoE)

Engineering Campus, Universiti Sains Malaysia, Seri Ampangan

14300 Nibong Tebal, Penang, Malaysia

Email: [redac01@usm.my](mailto:redac01@usm.my)

**Corresponding author**

Keng Yuen Foo (K.Y. Foo)

River Engineering and Urban Drainage Research Centre (REDAC)

Higher Institution Centre of Excellence (HiCoE)

Engineering Campus, Universiti Sains Malaysia, Seri Ampangan

14300 Nibong Tebal, Penang, Malaysia

Email : [k.y.foo@usm.my](mailto:k.y.foo@usm.my) (K. Y. Foo)

Tel: +6045996539; Fax: +6045996926


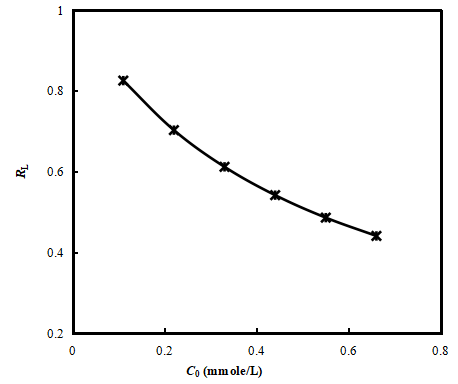


(a)


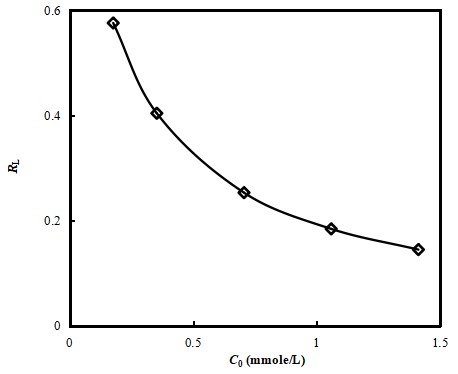


(b)

**Supplemental Figure 1.** Essential characteristic of the Langmuir isotherm model, separation factor, *R*_L_ as

a function of initial concentration for (a) ametryn and (b) metolachlor


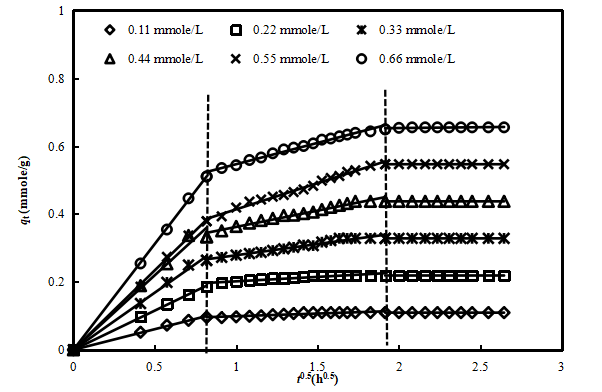
 (a)

***k*_p2_**

***k*_p1_**

***k*_p3_**


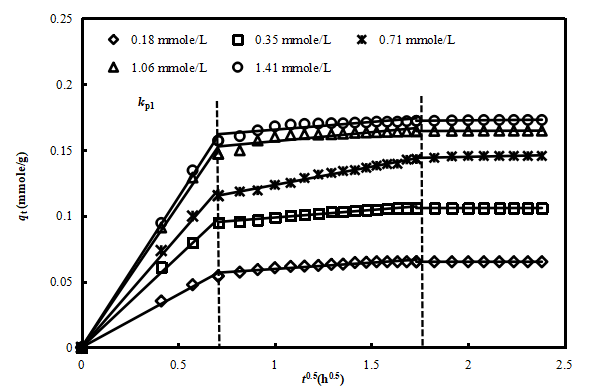
 (b)

***k*_p2_**

***k*_p3_**

**Supplemental Figure 2.** Intraparticle diffusion plots for the adsorption of (a) ametryn and (b) metolachlor onto

AB


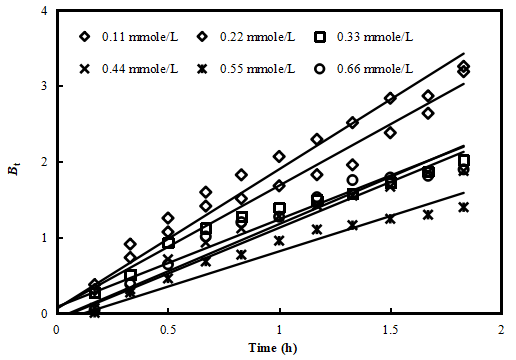


(a)


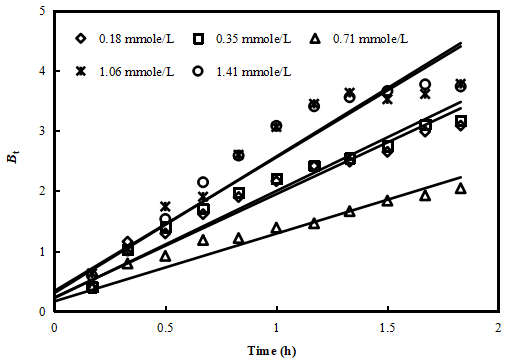


(b)

# Supplemental Figure 3. Plots of Boyd model for the adsorption of (a) ametryn and (b) metolachlor onto AB

# 30 °C


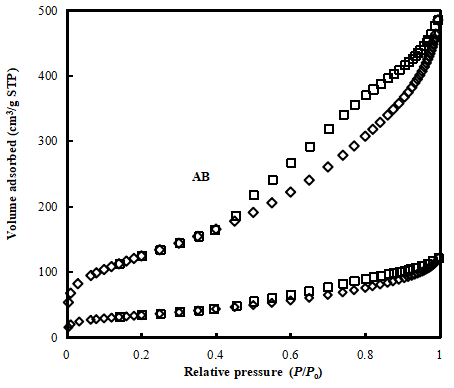


**Bentonite**

# Supplemental Figure 4. Nitrogen adsorption-desorption curves of bentonite and AB


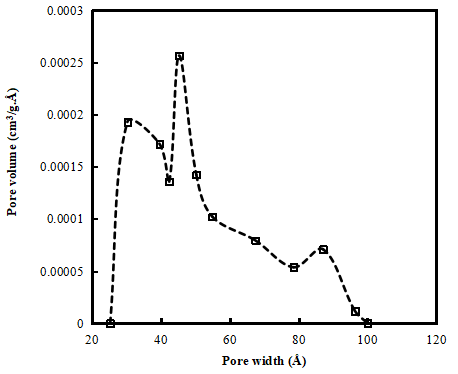


(a)


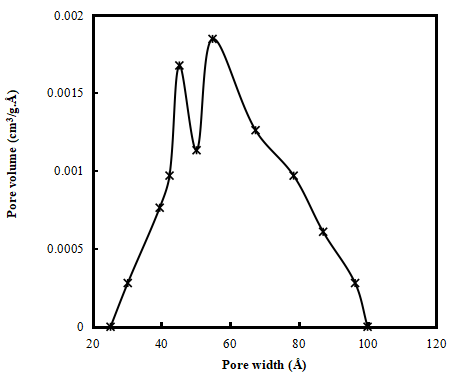


(b)

# Supplemental Figure 5. Pore-size distributions of (a) bentonite and (b) AB

**Supplemental Table 1.** Kinetic parameters for the adsorption of ametryn and metolachlor onto AB at 30 °C

| ***C*_0_**  **(mmole/L)** | ***q*_e,exp_**  **(mmole/g)** | **Pseudo-first-order kinetic model** | | | | **Pseudo-second-order kinetic model** | | | |
| --- | --- | --- | --- | --- | --- | --- | --- | --- | --- |
|  |  | ***q*_e,cal_ (mmole/g)** | ***k*_1_ (1/h)** | ***R*^2^** | **Δ*q* (%)** | ***q*_e,cal_ (mmole/g)** | ***k*_2_ (g/mmole h)** | ***R*^2^** | **Δ*q* (%)** |
| Ametryn |  |  |  |  |  |  |  |  |  |
| 0.11 | 0.110 | 0.104 | 7.061 | 0.996 | 6.00 | 0.117 | 44.432 | 0.998 | 6.36 |
| 0.22 | 0.219 | 0.189 | 5.596 | 0.984 | 13.70 | 0.241 | 18.340 | 0.998 | 10.05 |
| 0.33 | 0.328 | 0.284 | 4.995 | 0.959 | 13.42 | 0.346 | 12.005 | 0.999 | 5.49 |
| 0.44 | 0.437 | 0.364 | 4.465 | 0.913 | 16.71 | 0.461 | 8.302 | 0.999 | 5.49 |
| 0.55 | 0.547 | 0.477 | 3.998 | 0.984 | 12.80 | 0.593 | 3.959 | 0.996 | 8.41 |
| 0.66 | 0.655 | 0.603 | 4.718 | 0.989 | 7.98 | 0.699 | 4.834 | 0.999 | 6.71 |
| Metolachlor |  |  |  |  |  |  |  |  |  |
| 0.18 | 0.065 | 0.060 | 8.199 | 0.986 | 7.69 | 0.069 | 100.445 | 0.998 | 6.15 |
| 0.35 | 0.106 | 0.091 | 8.399 | 0.953 | 14.15 | 0.110 | 79.367 | 0.999 | 3.77 |
| 0.71 | 0.146 | 0.141 | 8.099 | 0.988 | 3.43 | 0.144 | 75.586 | 0.999 | 1.37 |
| 1.06 | 0.165 | 0.142 | 8.589 | 0.961 | 13.94 | 0.169 | 76.272 | 0.999 | 2.42 |
| 1.41 | 0.173 | 0.158 | 9.499 | 0.976 | 8.67 | 0.178 | 69.501 | 0.999 | 2.89 |

**Supplemental Table 2.** Intraparticle diffusion model parameters for the adsorption of ametryn and metolachlor onto AB at 30 °C

| ***C*_0_** | **Intraparticle diffusion model** | | | | | | | | |
| --- | --- | --- | --- | --- | --- | --- | --- | --- | --- |
| **(mmole/L)** | ***k*_p1_ (mmole/g h^1/2^)** | ***k*_p2_ (mmole/g h^1/2^)** | ***k*_p3_ (mmole/g h^1/2^)** | ***C*_1_** | ***C*_2_** | ***C*_3_** | **(*R*_1_)^2^** | **(*R*_2_)^2^** | **(*R*_3_)^2^** |
| Ametryn | | | | | | | | | |
| 0.11 | 0.122 | 0.016 | - | 0 | 0.083 | 0.110 | 0.996 | 0.880 | - |
| 0.22 | 0.231 | 0.026 | - | 0 | 0.176 | 0.219 | 0.999 | 0.852 | - |
| 0.33 | 0.337 | 0.066 | - | 0 | 0.213 | 0.328 | 0.993 | 0.979 | - |
| 0.44 | 0.440 | 0.096 | - | 0 | 0.266 | 0.437 | 0.981 | 0.982 | - |
| 0.55 | 0.468 | 0.154 | - | 0 | 0.260 | 0.546 | 0.999 | 0.989 | - |
| 0.66 | 0.624 | 0.130 | 0.010 | 0 | 0.421 | 0.643 | 0.999 | 0.957 | 0.4958 |
| Metolachlor | | | | | | | | | |
| 0.18 | 0.080 | 0.010 | - | 0 | 0.050 | 0.065 | 0.992 | 0.897 | - |
| 0.35 | 0.138 | 0.012 | - | 0 | 0.087 | 0.106 | 0.995 | 0.972 | - |
| 0.71 | 0.169 | 0.023 | 0.003 | 0 | 0.096 | 0.139 | 0.995 | 0.989 | 0.561 |
| 1.06 | 0.216 | 0.014 | 0.001 | 0 | 0.143 | 0.164 | 0.995 | 0.724 | 0.695 |
| 1.41 | 0.228 | 0.012 | 0.001 | 0 | 0.154 | 0.170 | 0.988 | 0.750 | 0.836 |

# Supplemental Table 3: Thermodynamics analysis for the adsorption of ametryn and metolachlor onto AB.

| **Pesticide** | **Δ*G*º (kJ/mole)** | | | **Δ*H*º (kJ/mole)** | **Δ*S*º**  **(J/mole *K*)** |
| --- | --- | --- | --- | --- | --- |
|  | **30 °C** | **40 °C** | **50 °C** |
| Ametryn | -12.74 | -11.13 | -10.04 | -42.08 | 196.14 |
| Metolachlor | -0.09 | 1.34 | 3.69 | -57.18 | -187.94 |

**Supplemental Table 4.** Surface physical properties of raw bentonite and AB

| **Properties** | **Bentonite** | **AB** |
| --- | --- | --- |
| BET surface area (m^2^/g) | 120.35 | 464.92 |
| Micropore surface area (m^2^/g) | 11.26 | 23.28 |
| External surface area (m^2^/g) | 109.08 | 441.64 |
| Langmuir surface area (m^2^/g) | 151.75 | 558.48 |
| Total pore volume (cm^3^/g) | 0.1550 | 0.2388 |
| Micropore volume (cm^3^/g) | 0.0171 | 0.0067 |
| Mesopore volume (cm^3^/g) | 0.1379 | 0.2321 |
| Average pore size (Å) | 46.67 | 63.48 |
